# Supplementary material for: Multi-level factors influencing HIV risk behaviors and oral PrEP use among Black and Latino men with heterosexual contact in New York City
Source: PLoS One. 2025 Jul 11;20(7):e0327677. doi: 10.1371/journal.pone.0327677 (PMC12250516; doi:10.1371/journal.pone.0327677)
Supplement: S1 File — (DOCX) [file pone.0327677.s001.docx]

**Pre-Interview Brief Survey**

1. *What is your race/ethnicity? White/Caucasian, Black/African American, Hispanic/Latinx, Asian, Other.**
2. *What is your gender? Transman, man, other*
3. *What describes your education level? Less than high school degree, High school degree, Technical/Associate degree, Some college, College, Some graduate school.*
4. *[Include question on sexual orientation]*

**SNAP In-depth Interview Guide**

*Thank you for answering the survey questions. I’d like to move on to the next part of the interview. You can think of this part of the interview as more of a conversation. I’ll be asking you a series of questions, and am interested in learning about your experiences, beliefs, and opinions in more details. There are no right or wrong answers, and if you don’t understand my questions, please let me know and I will try to clarify them.*

| **QUESTION** | **PROBE** |
| --- | --- |
| **Personal characteristics** | |
| Can you tell me a little about yourself? | - Household structure, employment, marital status - [If unemployed] how do you get by financially? |
| Do you have any health concerns? If so, which ones are the most important to you? | - Mental health, chronic disease, STIs/HIV, family planning, weight loss, health insurance, **substance use** |
| Do you have any non-health concerns? Which ones do you spend the most time worrying about? | - Finances, employment, education, childcare, housing, legal (documentation status) |
| **Sexual relationships** | |
| Can you tell me about your current (or last) sexual/romantic relationship(s)? | - What was the gender of your partner? - What was the nature of the relationship? (Monogamous? Casual? Long-term?) - Did you feel safe in this relationship? |
| What do you do to protect yourself against STIs or HIV during your sexual encounters? | - What methods have you or your partner used? - What methods have worked for you or your partner the best? Why? - What methods haven’t worked for you or your partner so well?   Why? |
| Violence | - Have you experienced any violence or abuse from family members or romantic partners in the past that we haven’t talked about? - Transmen only: Have you experienced any violence or abuse because of your trans identity or appearance? |
| Sex Work | - Have you ever provided sexual services and received money in return?   IF YES: is this something that you currently engage in? |
| **Facilitators and Barriers regarding PrEP use** | |
| Perceived HIV risk: Do you feel that you’re at risk for HIV? Why or why not? | - Do you ever discuss sexual histories with your partners? - Do you know anyone with HIV? - Have you ever had an STI? |
| My understanding is that you were prescribed PrEP but did not refill your PrEP medication. | - Do you know any other men who are taking PrEP? - How do you think other people feel about PrEP? (probe: do you feel that PrEP is more appropriate for gay men?) - How much of the prescribed dosage did you take? - What are your main reasons for stopping PrEP? Probes: - side effects, - not remembering to take it everyday, - doesn’t like taking pills, - partner not supportive, accused of not being faithful/cheating, - PrEP/HIV/gay stigma, - Decreased motivation for using condoms, - difficulty in refilling prescription, - no longer perceives HIV risk (e.g., Entered a stable, monogamous relationship) - other life priorities - COVID-related concerns   Would you consider going on PrEP again in the future? |

**Final thoughts**

- Is there anything else you would like to share that we have not talked about?
- Do you have any questions for me?

***Thank you!***
