# Supplementary material for: Multi-level factors influencing HIV risk behaviors and oral PrEP use among Black and Latino men with heterosexual contact in New York City
Source: PLoS One. 2025 Jul 11;20(7):e0327677. doi: 10.1371/journal.pone.0327677 (PMC12250516; doi:10.1371/journal.pone.0327677)
Supplement: Attachment — Submitted filename: Reviewers comments.docx [file pone.0327677.s002.docx]

Reviewer’s comments_PONE-D-24-18209

Major

Minor

Definition of prep (this could be daily or event driven
