# Supplementary material for: Multi-level factors influencing HIV risk behaviors and oral PrEP use among Black and Latino men with heterosexual contact in New York City
Source: PLoS One. 2025 Jul 11;20(7):e0327677. doi: 10.1371/journal.pone.0327677 (PMC12250516; doi:10.1371/journal.pone.0327677)
Supplement: Attachment — Submitted filename: Reviewer 2 comments_PONE-D-24-18209.docx [file pone.0327677.s003.docx]

**Reviewers’ comments: PONE-D-24-18209**

This is an interesting study, reacting to the increasing new HIV infections among Black and Latino men with heterosexual contact in New York City by gaining an understanding of, and giving important insights into the multi-level **factors that influence** HIV risk behaviours and oral PrEP use among this population, hence contributing to the limited literature on the factors that influence HIV risk behaviours and PrEP use among this population. The study provides an understanding of how HIV risk and PrEP use are perceived among this Black and Latino population, which findings may facilitate HIV prevention efforts in this population if the authors revise the paper to make it more robust, with a clearer flow of the findings.

Below are some comments:

Overall, the introduction section is well written, with evidence of enough literature to support the authors claim and a clear aim of the study hence giving a concrete ground for the study.

**Major issues**

Methods

I suggest that the authors consider re-organising the different sections of the methods to give a clear flow to the processes.

ii) From ln 80, in the methods section, the authors indicate participants/study participants interchangeably, while there is a need to standardize this, the authors need to clearly indicate if the consenting and recruitment that started June 19 was for the larger trial or qualitative study participants. Also briefly explain the ‘on-site navigators’ (ln 84), were these health care providers/part of the research team and the ‘longitudinal care’ on line 87; whether this care lasted for the duration of the trial, the authors may indicate that duration of the trial.

ii) Ln 92 shows that participants were interviewed at any time point; authors need to indicate what influenced the timing for the interview and at what point during the larger trial these interviews were conducted and what influenced this and whether it was a one-time-point or repeat interviews (this bit could go under data collection)

iii) Ln 97, the sub section ‘Procedures’, I suggest that the authors be more specific and revise to ‘Data collection’ since the preceding and succeeding sub sections are part of the procedures. The authors could give the section a better flow by starting with when the interviews started and ended.

iv) Ln 112 on approvals, I suggest that you include information on consent since the written consent indicated in ln 80 seem to be for the larger trial. If that is the case, indicate if that catered for the qualitative component or participants were reconsented for the qualitative study. If they reconsented, was this written or verbal? For those interviews conducted on phone (ln 104), how was the consenting process handled?

V) I suggest that the authors indicate a clear chronological flow of the analysis as per the steps of thematic analysis (much as other analysis approaches were integrated). Ln 115 shows the coding of interviews, but I suggest the authors indicate how they arrived at the codes. Also, ln 117 to 118, authors need to clearly indicate how they arrived at the themes. Was this directly from reading the transcripts or did they group/categorise codes to get themes?

Results

vi) Some of the sub-themes do not seem to speak directly or to match the main themes. For example, line 187, although the text below the sub-theme ‘HIV-related stigma’ matches/relates with the main theme, the sub-theme name does not. I suggest that you merge it with ln 144 ‘self-perception of sexual risk’. Also ln 216 ‘Understing of PrEP’, this would better suit under theme 2, ln 242 on ‘Attitudes towards PrEP…’

Discussion

vii) Ln 506 to 507, the claim that participants found solidality with partners who shared similar pasts’ does not seem to appear anywhere in the results

**Minor issues**

viii) Ln 55 in the introduction, the authors explain oral PrEP as a medication taken daily to prevent HIV. With the current efforts to promote event-driven/on-demand PrEP, I suggest that the authors indicate both oral PrEP options.

ix) I suggest that the authors edit the typos and grammatical errors [in the entire] manuscript to improve the flow. The authors may also consider putting an explanation in brackets against the quotes where the language/dialect may not be clear to any reader e.g ln 184-186.

x) Line 136 to 137 (the table); I would suggest that given the small number of participants, would the authors consider expanding the table to indicate characteristics for each participant. This will give a clear picture of the participants.

xi) Ln 545 in the acknowledgments, the authors know better, but it would be good to acknowledge the participants and even other members at the clinic who supported the study, for example, the on-site navigators who supported in identifying the participants
