# Supplementary material for: Multi-level factors influencing HIV risk behaviors and oral PrEP use among Black and Latino men with heterosexual contact in New York City
Source: PLoS One. 2025 Jul 11;20(7):e0327677. doi: 10.1371/journal.pone.0327677 (PMC12250516; doi:10.1371/journal.pone.0327677)
Supplement: Attachment — Submitted filename: Response to Reviewers_5.20.25.docx [file pone.0327677.s005.docx]

**REVIEWER #1**

**Overall Comments**

1. Potential bias of the researchers leading to concerns about how data was collected and analyzed.

We agree that bias is always a concern in regards to qualitative data collection and analysis, and have attempted to minimize such bias through a rigorous, team-based approach and have added this in the Data Analysis section:

“To address potential researcher bias in data collection and analysis, we employed reflexive practices throughout the study. During data collection, the use of a semi-structured guide allowed for consistency between interviewers while allowing participants to expand upon their own perspectives. Throughout data collection and analysis, team members engaged in regular debriefs and peer reviews of coding decisions to reflect on how positionality may influence the interpretation of responses and ensure that interpretations remained grounded in the data.

We also explicitly looked for and reframed stigmatizing language and/or language that feels laden with bias against participants.

2. Unclear if the sample size was sufficient to reach saturation on these primary themes among a heterogenous sample of LEP and English-speaking Black and Latino men,

We agree that the sample consists of a heterogenous sample and attempted to only highlight themes that reached saturation. The following statement was added in our discussion:

“While the smaller sample size may not fully capture the full range of experiences given the heterogeneity of LEP and English-speaking Black and Latino men, the depth and consistency of interview responses suggest that thematic saturation was achieved.”

3. Characterization of interviewee participants is lacking leading to low generalizability of results.

We included additional information in our Table 1 to provide the audience with additional characterization of our sample as allowed by in the consent document. It should be noted that the goal of qualitative research is not necessarily to generalize to a larger sample, but rather to gain a nuanced understanding directly from participants, particularly from those who are under-researched.

4. Missed opportunities here to extend the research especially regarding how either of these groups of heterosexual men (Latino, Black, LEP) view PrEP in their individual contexts.

Given our smaller sample size, we decided to focus on extending our research findings to common patterns we saw across our sample of Black and Latino men with heterosexual contact. We recognize this as an important starting point for understanding how PrEP is perceived within these communities. We hope our multiple calls to action regarding future research with larger, more segmented samples will result in research that is more tailored to individual contexts.

**Abstract**

- Background: Make clear that this is a heterogenous sample of LEP men and English-speaking men. The way it is written currently led me to believe on first read that the sample was all LEP black and Latino men but after reading to the methods within the paper, I now realize it is a mix of English-speaking and LEP men. I think this should be clear as some of the barriers faced to PrEP care for LEP men likely differ from English-speaking men.

We made this clearer in the abstract:

“This study sought to understand factors influencing HIV risk behaviors and oral PrEP use among a heterogenous sample of low income, limited English proficient (LEP) or English-speaking, Black and Latino men with heterosexual contact in New York City (NYC).”

- Inclusive language, “substance use problems” in results section should just be substance use or negative consequences due to substance use depending on what we are trying to get at here.

Thank you for pointing out that this language can be stigmatizing. We changed this to “substance use.”

- Conclusion should be more concrete towards the results and not a call or discussion of implications.

We strongly believe that having an implication of discussion/call to action is important given our research findings and have thus recategorized the Conclusion section as a “Discussion” section to make this clearer.

**Introduction**

- Page 3, lines 55-64, it might be helpful to include the clinical guidelines for PrEP for heterosexual men or people in general. US Public Health Service: PREEXPOSURE PROPHYLAXIS FOR THE PREVENTION OF HIV INFECTION IN THE UNITED STATES – 2021 UPDATE, A CLINICAL PRACTICE GUIDELINE ([cdc.gov](http://cdc.gov/))

We agree that it would be helpful to lay-out what the clinical guidelines for PreP for heterosexual men are and have added the following information:

“In the same year, the CDC recommended the administration of PrEP to 624,000 heterosexual individuals in the U.S. with indications for PrEP including having vaginal or anal sex in the past six months with an HIV positive partner, or with one or more sexual partners of unknown HIV status, or having been diagnosed with a sexually-transmitted illness in the past six months”

- Page 3 lines 65-68, please make clear here that a similar shared characteristic among the population you interviewed were LEP.

We hope the clarification in the abstract has made it clearer that this is a heterogeneous sample that includes both LEP and English-Speaking individuals.

- Page 4 line 72, please make clear here if the entire interview sample are LEP or if it is a heterogenous group of LEP heterosexual men and English-speaking men.

Likewise, we have added clarification in the introduction to make this clear:

“The study elicits voices from a heterogenous sample that includes both English speakers and LEP Spanish-speaking men, as these are the most commonly spoken languages among Black and Latino men in NYC.”

- Page 4 72-73 “U.S. States federal government” is awkward phrasing. Are you saying the State government of NY or the federal government of the United States.

We agree that this phrasing is awkward or may add to confusion, and have changed it to:

“Given the U.S. federal government’s commitment to reducing new HIV infections by 90% by 2030, as well as the continued roll-out of newer long-acting injectable PrEP, it is imperative that HIV prevention efforts equitably understand factors impacting HIV prevention efforts among at-risk, and underserved populations

**Method**

- The inclusion/exclusion criteria for both the parent study and the qualitative study presented in this manuscript should be stated much more clearly. For example, Page 4, Line 87-88 “indicated for PrEP” does this mean they were approached about PrEP and seemed interested or was this that they seemed to meet the clinical guidelines to be prescribed PrEP?. Additionally Page 4, Line 90-92: “…, engaged in heterosexual contact at the time of recruitment,..” Within what time period?

We clarified the language to include that they were approached about PrEP according to clinical guidelines:

“Participants for the larger clinical trial were eligible if they were HIV negative and met the clinical guidelines for PrEP prescription as assessed by the Sexual Health Clinic providers utilizing the Centers for Disease Control & Prevention guidelines”

Additionally, we clarified that individuals were eligible if they reported heterosexual contact at their last sexual encounter:

“Participants of the qualitative study were eligible if they identified as a cisgender man, as Hispanic/Latino or Black, engaged in heterosexual contact at their last sexual encounter, and spoke English or Spanish.”

- Some of the information about recruitment for the larger study and this study are unclear. For example, Page 4, lines 82-86, participants of the larger trial were recruited from various departments within the hospital. Then the next sentence says they were identified through the emergency room. Which was it? Clarify.

We apologize for this confusion and have clarified that individuals were recruited from emergency departments.

- As many of these participants may have been at different places within their larger trial participation and that this trial participation seems to have convinced at least some to try or get on PrEP, it seems important to detail where each participant was on the trial continuum. Should be added to a Table 1.

While we have not included specific information on where individuals were in the trial, we included information regarding whether individuals were on PrEP at the time of the interview, when they had been first prescribed PrEP, and the date since the last PrEP refill in Table 1. We were unable to include any other additional information about participants along the trial continuum as our consent forms do not specify being able to elicit information beyond PrEP information from the larger trial.

- Include the interview guide as an appendix

We have attached the English version of the interview guide as an appendix.

- Data analysis: Was the codebook closed or open? Could new codes be added during the coding process? If they were, how did you handle it?

We included more description of the data coding process:

“The codebook was treated as an open, living document, and codes were added and redefined as new themes emerging from the data. Decisions to add codes were discussed regularly among the coders, and earlier transcripts were revisited when new codes were added to ensure uniform application of codes across the transcripts”

- What did the study team do to address and mitigate their own biases within the data collection and analysis phases of this study?

The study team consistently engaged in reflexive practices, and we agree that this is especially important to explicitly mention due to the nature of our research. We added the following statement:

“To address potential researcher bias in data collection and analysis, we employed reflexive practices throughout the study. During data collection, the use of a semi-structured guide allowed for consistency between interviewers while allowing participants to expand upon their own perspectives. Throughout data collection and analysis, team members engaged in regular debriefs and peer reviews of coding decisions to reflect on how positionality may influence the interpretation of responses and ensure that interpretations remained grounded in the data.”

- Is there any information available as to how study investigators decided thematic saturation had been reached?

We added how we determined thematic saturation in the procedures section:

“Interviews were conducted until reaching thematic saturation, which involved team members engaging in an iterative review of the data during the coding process to ensure no new themes or concepts were emerging, and existing codes sufficiently captured the responses across the participants.”

**Results**

- It’d be nice to know where each participant was on the trial continuum if that information is available. I think it’s important contextual information to characterize participants, especially if any of them got on PrEP during the course of the trial, as stated in previous comment.

We included information regarding PrEP prescription, and we were unable to include any other additional information about participants along the trial continuum as our consent forms do not specify being able to elicit information beyond PrEP information from the larger trial.

- Is there more demographic information available for these participants? For example, Page 8, Lines 193-195 references they aren’t sharing needles. Do you have any further information to better characterize this group of black/and Latino English speaking and LEP men like percentage that are PWID, HIV risk behaviors, others to better characterize participants?

Likewise, we have included as much information as we can as specified in our consent and IRB application process. We hope the addition of PrEP prescription information adds a little bit more context.

- Further, is there any information as to how many potential participants were approached yet refused? This is important for helping the reader understand the generalizability of the sample.

We used a convenience sampling approach, primarily driven by who the research team thought would be willing to participate and do not have any information regarding how many participants were approached yet refused. We recognize that this may add to some issues around generalizability, but we again believe that the purpose of qualitative research is not generalizability but to gain a nuanced understanding directly from participants, particularly from those who are under-researched.

- Page 7 Lines 166-172: Some of this feels like participant blaming. Is there a better framing maybe more towards low levels of health literacy and knowledge about how their own behaviors influence risk?

We reframed these findings to provide broader social context to avoid participant blaming:

“The participant appears to externalize his perception of HIV risk by focusing more on trust in his partners and uncertainty about their sexual histories, reflecting a broader dynamic in which risk assessment is shaped by limited access to accurate sexual health information and low levels of health literacy around his own sexual behaviors. To this end, this participant did express some levels of perpetual distrust towards his intimate partners outside of his personal assessment of sexual transmission risk with a given sexual partner—suggesting that personal judgment alone is insufficient in fully trusting a sexual partner.”

- Page 9 Line 212 to 215: This again reads like blaming the participant but there are many contextual reasons that this happens that is beyond the participants control and is rooted in the contextual experience within which this man lives. Calling this misperception “misguided” feels like it belittles the contextual factors within which Black and Latino men exist like masculinity which, despite its effect on women comes with its own negatives for men. This leads to questions about bias influencing the study.

We agree again that we always need to be contextualizing findings in terms of the societal forces that shape experiences for individuals. We have modified the language in accordance to this:

“This characterization of individuals with HIV as visibly “sick” perpetuates the erroneous narrative that individuals with HIV are unable to live long, healthy lives. This perception is deeply embedded in broader sociocultural narratives that influence how risk is understood and may lead to a misperception of an individual’s own sexual risk level, when in reality many individuals with HIV, even when untreated, may present as relatively healthy early on in their infection.”

- Page 9 Lines 216-227 illustrates why there needs to be further characterization of these men. Were the majority of participants on PrEP after the intervention? I think this holds implications for the understanding of this research.

Since the intervention was ongoing, we do not have information regarding whether the majority of participants were on PrEP after the intervention.

- Page 12 Line 284 – 286: Please find an alternative language to “double standards”.

We have described double standards in a clearer way to avoid confusion and misrepresenting what we mean:

“Men conformed to social norms around hegemonic masculinity through misogynistic characterizations of women and inconsistent expectations placed on women versus men regarding multiple sexual partnerships.”

- Page 12 Line 294-297: This quote does not seem to go with the written passage above it. Seems to me this person is saying that you never know who you can trust, even your wife or husband can be in an adulterous relationship. I don’t read this as viewing women as “implicitly adulterous”.

We agree that the quote does not align well with the theme and may be extrapolating inferences outside of what is written. We have decided to remove this quote.

- The authors should adjust their tone when discussing their participants who trusted them enough to participate in interviews and share their stories. The current tone feels laden with bias against participants who agreed to be interviewed.

We strongly agree with you that language should be used more precisely, and individual behavior must be contextualized with broader societal norms/structural inequities that give rise to these phenomena. While we had attempted to do this, there were several instances where the tone could be interpreted in a biased manner and have thoroughly examined situations in which we could avoid blaming language in accordance to the reviewer’s suggestions. Additionally, we organized this paper by starting off with individual factors and expanding out into broader societal factors to ensure that we ultimately hold structural factors responsible for individual behaviors.

Thank you for pointing this out and ensuring that the manuscript findings can be communicated in an appropriate way.

**Discussion**

- The last paragraph of this discussion would be strengthened by providing key conclusions/summary of findings.

We added some of our key themes from the results in the concluding paragraph:

“Particularly, our analysis highlights how hegemonic masculinity and experiences across the life course shape the role of communal and societal norms in influencing men’s HIV risk behaviors and oral PrEP usage. Themes around mistrust, gendered power dynamics, mixed sexual networks, and structural barriers underscore the complexity of HIV prevention within this population.”

**REVIEWER #2**

Reviewer #2: Like I indicated in my comments, the authors need to edit the language including the grammar, typo errors and punctuation as well as explaining some of the quotes, which are not clear. I suggested putting an explanation in brackets again the quotes that are not clear.

Thank you for the comments. We have attempted to review the manuscript and ensure that there are no grammar, typos, and punctuation errors. Additionally, we have addressed the confusion around some of the quotes listed in your feedback on the results (see below).

**Methods**

- I suggest that the authors consider re-organising the different sections of the methods to give a clear flow to the processes.

We have reorganized the methods sections in the way that the reviewer suggests.

- From ln 80, in the methods section, the authors indicate participants/study participants interchangeably, while there is a need to standardize this, the authors need to clearly indicate if the consenting and recruitment that started June 19 was for the larger trial or qualitative study participants. Also briefly explain the ‘on-site navigators’ (ln 84), were these health care providers/part of the research team and the ‘longitudinal care’ on line 87; whether this care lasted for the duration of the trial, the authors may indicate that duration of the trial.

We have clarified the use of language of “participants” for the larger clinical trial, and “study participants” for the specific qualitative study that we are presenting in the manuscript. We have included several changes to the participant section to enhance clarity around the consenting procedures for the larger trial vs. qualitative study, on-site navigators, and longitudinal care:

“Participants in the larger clinical trial were enrolled from June 19, 2019 through January 21, 2022. [Blinded for review] Hospital is part of a larger network of safety net hospitals providing care for underserved New Yorkers. The intervention component of the larger clinical trial involved recruiting individuals from the emergency department within the hospital into the Sexual Health Clinic with the assistance of on-site navigators. On-site navigators, who are part of the healthcare team and coordinate care for patients, reviewed the electronic health record of individuals admitted to the emergency room and screened for those who tested positive for a sexually transmitted infection.”

- Ln 92 shows that participants were interviewed at any time point; authors need to indicate what influenced the timing for the interview and at what point during the larger trial these interviews were conducted and what influenced this and whether it was a one-time-point or repeat interviews (this bit could go under data collection)

Interviews were collected on an ongoing basis after the end of the trial, around May 2023. which occurred between June 2019 – Jan 2022.

- Ln 97, the sub section ‘Procedures’, I suggest that the authors be more specific and revise to ‘Data collection’ since the preceding and succeeding sub sections are part of the procedures. The authors could give the section a better flow by starting with when the interviews started and ended.

We agree that this section applies more to data collection and have renamed the header. We have already included that the interviewers began in October 2021 and ended in December 2022:

“Interviews were conducted between October 2021 and December 2022.”

- Ln 112 on approvals, I suggest that you include information on consent since the written consent indicated in ln 80 seem to be for the larger trial. If that is the case, indicate if that catered for the qualitative component or participants were reconsented for the qualitative study. If they reconsented, was this written or verbal? For those interviews conducted on phone (ln 104), how was the consenting process handled?

We added consent specifically to the interviews in the procedures/data collection sections:

“Written informed consent was obtained prior to the interview (i.e., participants were reconsented).”

- I suggest that the authors indicate a clear chronological flow of the analysis as per the steps of thematic analysis (much as other analysis approaches were integrated). Ln 115 shows the coding of interviews, but I suggest the authors indicate how they arrived at the codes. Also, ln 117 to 118, authors need to clearly indicate how they arrived at the themes. Was this directly from reading the transcripts or did they group/categorise codes to get themes?

We have added more explicit steps of how we conducted analysis and arrived at the codes.

“Data were iteratively analyzed using an inductive process integrating narrative and thematic approaches [17]. The team first familiarized themselves by reading and re-reading the interview transcripts. Two members of the study team (first and second author; blinded for review) independently conducted initial coding of a subset of transcripts to capture concepts that emerged directly from participant narratives. Subsequently, these initial codes were compiled into a preliminary codebook developed iteratively by a team of two coders. This codebook was treated as an open, living document, and codes were added and redefined as new themes emerging from the data. Decisions to add codes were discussed regularly among the coders, and earlier transcripts were revisited when new codes were added to ensure uniform application of codes across the transcripts. Reliability of coding was ensured through frequent discussions and consensus between coders to achieve inter-coder reliability [18]. After reading transcripts multiple times, the study team compiled several themes and subthemes across participants, utilizing each participants’ narratives to ensure that responses were grounded in their specific contexts. Themes were grouped by frequency and conceptual relevance across the interviews.”

**Results**

- Some of the sub-themes do not seem to speak directly or to match the main themes. For example, line 187, although the text below the sub-theme ‘HIV-related stigma’ matches/relates with the main theme, the sub-theme name does not. I suggest that you merge it with ln 144 ‘self-perception of sexual risk’. Also ln 216 ‘Understing of PrEP’, this would better suit under theme 2, ln 242 on ‘Attitudes towards PrEP…’

We respectfully believe that the sub-theme HIV-related stigma represents the findings below it, as participants underscored negative attitudes, beliefs, and judgements about individuals living with HIV that are consistent with this concept and inter-related with conceptualization of sexual risk. We agree that “Understanding of PrEP” would fit better under the Attitudes towards PrEP section and have moved this.

**Discussion**

- Ln 506 to 507, the claim that participants found solidarity with partners who shared similar pasts’ does not seem to appear anywhere in the results

We changed the language to be more aligned with the quote on lines 395-398 (in the previous manuscript version):

“Participants directly linked their sexual trauma and attitudes towards relationships, noting that such experiences allowed them to be more empathetic and sensitive to similar traumas experienced by potential partners and women.”

**Minor issues**

- Ln 55 in the introduction, the authors explain oral PrEP as a medication taken daily to prevent HIV. With the current efforts to promote event-driven/on-demand PrEP, I suggest that the authors indicate both oral PrEP options.

We have added information regarding on-demand PrEP as an option for oral PrEP:

“Low uptake of oral pre-exposure prophylaxis (PrEP), a highly effective medication taken daily or on-demand (i.e. two pills at least two hours before sex, one pill twenty four hours after the first dose, and one pill forty eight hours after the first dose) to prevent HIV acquisition among HIV-negative individuals, may also contribute to higher risk of HIV transmission among Black and Latino/a populations.”

- I suggest that the authors edit the typos and grammatical errors [in the entire] manuscript to improve the flow. The authors may also consider putting an explanation in brackets against the quotes where the language/dialect may not be clear to any reader e.g ln 184-186.

We have reviewed the entire transcript to ensure that there are no typos and grammatical errors. Additionally, we have included brackets in quotes for some sections of quotations that could use more quality, for example:

“First of all, [I stay] monogamous, safe. Basically abstinence, we don’t really engage [in sex]. I don’t go outside. I don’t do anything really, as far as that’s concerned”

- Line 136 to 137 (the table); I would suggest that given the small number of participants, would the authors consider expanding the table to indicate characteristics for each participant. This will give a clear picture of the participants.

We were unable to include any other additional demographic information about participants as our consent forms do not specify being able to elicit information beyond PrEP information and the already included demographic characteristics from the larger trial.

- Ln 545 in the acknowledgments, the authors know better, but it would be good to acknowledge the participants and even other members at the clinic who supported the study, for example, the on-site navigators who supported in identifying the participants

Thank you. We agree that an acknowledgement thanking the community of participants and on-site navigators who supported the participants and study would be good to include:

“We express our deepest gratitude to the participants who shared their time and experiences with us. We would also like to thank the on-site navigators and clinic staff who supported participant recruitment and engagement throughout the study.”
